# Jaceosidin Induces Apoptosis in U87 Glioblastoma Cells through G2/M Phase Arrest

**DOI:** 10.1155/2012/703034

**Published:** 2011-12-19

**Authors:** Muhammad Khan, Bo Yu, Azhar Rasul, Ali Al Shawi, Fei Yi, Hong Yang, Tonghui Ma

**Affiliations:** ^1^Membrane Channel Research Laboratory, Northeast Normal University, Changchun 130024, China; ^2^College of Life Sciences, Liaoning Normal University, Dalian 116029, China; ^3^Second Clinical Hospital, Jilin University, Changchun 130041, China

## Abstract

*Artemisia argyi* is a widely used medicinal plant in China. The present study was designed to identify the bioactive constituents with antiglioma activity from leaves of *Artemesia argyi*. A bioactivity guided approach based on MTT assay for cells growth inhibition led to the isolation of a flavonoid, “jaceosidin” from ethanol extract of leaves of *Artemesia argyi*. The growth inhibitory effect of jaceosidin was explored using flow cytometry and Western blot studies. Our results showed that jaceosidin exerts growth inhibitory effect by arresting the cells at G2/M phase and induction of apoptosis. Furthermore, our study revealed that induction of apoptosis was associated with cell cycle arrest at G2/M phase, upregulation of p53 and Bax, decrease in mitochondrial membrane potential, release of cytochrome c, and activation of caspase 3. This mitochondrial-caspase-3-dependent apoptosis pathway was confirmed by pretreatment with caspase 3 inhibitor, Ac-DEVD-CHO. Our findings suggested that jaceosidin induces mitochondrial-caspase-3-dependent apoptosis in U87 cells by arresting the cell cycle at G2/M phase.

## 1. Introduction

The genus *Artemisia*, belonging to the Compositae family, comprises around 500 species which are widespread throughout the world. *Artemisia* plants are important medicinal plants and have long been used in Chinese traditional medicines (TCM) to treat microbial infections, inflammatory diseases, diarrhea, gastric ulcer, malaria, hepatitis, cancer, and circulatory disorders [1–3].

Several phytochemical studies conducted on *Artemisia* plants have revealed the presence of coumarins, glycosides, sterols, polyacetylenes, monoterpenes, triterpenes, sesquiterpene lactones, flavonoids, polysaccharides, and essential oils [1, 3, 4]. Among flavonoid constituents, eupatiline, and jaceosidin got enormous attention due to their broad spectrum pharmacological activities including antiulcer, antiallergic, antidiabetic, antimutagenic, antiproliferative, antiinflammatory, antioxidative, and anticancer activities [5]. 

So far, jaceosidin has been reported to exhibit only a few chemoprevention-related pharmacological activities such as inhibition of COX-2 and MMP-9 in human mammary epithelial cells, suppression of E6 and E7 oncoproteins of HPV 16, induction of apoptosis in ras-transformed human breast epithelial cells and human ovary cancer cells [6–9]. However no information is at present available on the chemopreventive potential of jaceosidin on gliomas.

In the present study, we have isolated and purified jaceosidin from the leaves of *Artemisia argyi *by high-performance liquid chromatography (HPLC) and high-speed countercurrent chromatography (HSCCC). Furthermore, we investigated that the induction of apoptosis, mediated by jaceosidin in U87 glioma cells, evidenced by Bax activation, mitochondrial cytochrome c release and caspase 3 activation, was associated with cell cycle arrest at G2/M phase. Inhibition of caspase 3 did not prevent the G2/M phase arrest. These findings suggest that jaceosidin induced apoptosis is resulted from mitotic arrest.

## 2. Materials and Methods

### 2.1. Materials

The dried leaves of *Artemisia argyi* were purchased from Yunnan Qiancaoyuan Medicinal Plant Company (Yunnan, China) and identified by associate Professor Diao Yunpeng (Dalian Medical University, Dalian, China). Jaceosidin was isolated with 99.5% purity from leaves of *Artemisia argyi*. All the chemicals for extraction process were purchased from Honeywell Burdick & Jackson (USA). Dulbecco's Modified Eagle's Medium (DMEM) culture medium, Roswell Park Memorial Institute medium (RPMI 1640), fatal bovine serum (FBS), Propidium iodide (PI), RNase A, Rhodamine 123, [3-(4,5-Dimethylthiazol-2-yl)-2,5-Diphenyltetrazolium Bromide] (MTT), calcein acetoxymethyl-ester (calcein AM); and dimethyl sulfoxide (DMSO) were purchased from Sigma (Beijing, China). Annexin V-FITC Apoptosis Detection Kit, reactive oxygen species assay kit, and caspase 3 inhibitor, Ac-DEVD-CHO, were purchased from Beyotime (Shanghai, China). Antibodies specifice to p53, bax, cytochrome c, and caspase 3 were purchased from Beyotime (Shanghai, China) while *β*-actin and secondary antibodies were purchased from Santa Cruz (USA).

### 2.2. Extraction and Isolation

Dried leaves of *Artemisia argyi* were extracted with 95% ethanol. The ethanol extract was evaporated to dryness by using a rotor evaporator machine at 90°C under reduced pressure to get crude extract. The crude extract was then dissolved in 80% methanol and fractionated into 80 fractions by using 0–90% methanol gradient on preparative high-performance liquid chromatography (HPLC) equipped with 2489 UV/visible detector, 2495 separation module and 2767 collector (WATERS, USA). Each fraction was dried and dissolved in DMSO (10 mg/mL). All fractions were then screened against U87 glioblastoma cells at final concentration of 100 *μ*g/mL to identify bioactive fraction. After the identification of bioactive fraction, the crude extract was then extracted with different solvents to get maximum solubility of target peak (bioactive fraction) and further purified by high-speed countercurrent chromatography (HSCCC).

### 2.3. HSCCC Apparatus

The HSCCC instrument employed in the present study is TBE-300 HSCCC (Tauto Biotechnique Company, Shanghai, China) with three multilayer coil separation columns connected in series (ID of the tubing = 1.8 mm, total volume = 280 mL). The revolution radius was 5 cm, and the *β* values of the multilayer coil varied from 0.6 at the internal terminal to 0.8 at the external terminal. The speed of revolution of the apparatus could be regulated with a speed controller within the range 0–1000 rpm. The solvent was infused into the column using a TBP-50A pump (Tauto Biotechnique Company, Shanghai, China), and the eluent was continuously monitored by connecting the tail outlet of the coiled column to TBD-23UV (Tauto Biotechnique Company, Shanghai, China) system.

### 2.4. Preparation of the Two-Phase Solvent System and Sample Solution

The solvent system of n-hexane/ethyl acetate/methanol/water was optimized to the ratio of 1 : 1 : 1 : 3 (v/v/v/v). The two-phase solvent system was prepared by adding the solvents to a separation funnel according to the volume ratios and fully equilibrated by shaking repeatedly at room temperature. The upper and lower phases were separated shortly before use and degassed by sonication for 30 min. 30 mg of bioactive fraction were dissolved in 10 mL lower phase.

### 2.5. HSCCC Separation Procedure

The whole procedure was carried out as follows: the coil column was first filled with the upper phase of n-hexane/ethyl acetate/methanol/water (1 : 1 : 1 : 3 by volume) at a flow rate of 10 mL/min. The apparatus was rotated at 800 rpm, while the lower phase was pumped into the column at a flow rate of 2.0 mL/min. After the mobile phase front emerged and hydrodynamic equilibrium was established in the column, 10 mL sample solution was injected through the injector. The effluent from the outlet of the column was continuously monitored at 254 nm by UV–Vis detector and N2000 Station. Each peak fraction was manually collected according to the chromatogram and evaporated under reduced pressure. The residue was dissolved in acetonitrile for subsequent HPLC analysis.

### 2.6. HPLC Analysis and Identification of HSCCC Peak Fraction

The peak fraction obtained by HSCCC was analyzed by analytical reversed-phase (RP) HPLC (WATERS 2695). The column used was XTerra MS C18 (5 *μ*M, 2.1 × 150 mm). The mobile phase composed of acetonitrile (A) and 0.2% formic acid (B) was eluted with linear gradient elution (A: 0–25 min, 10–90%; 25–30 min, 90–100%). The injection volume was 10 *μ*L and flow rate was 0.2 mL/min. The effluent was monitored at 342 nm. Identification of the peak was carried out by liquid chromatography/mass spectrometry (LC/MS) and nuclear magnetic resonance (NMR). Mass spectrometry was performed using a Quattro Micro (Waters, USA) scanning from 50–1000 m/z. ^1^H and ^13^C NMR spectra were recorded on Bruker Avance 500 spectrometer (Bruker Optics-Beijing, China) operating at 500 MHz for ^1^H and 100 MHz for ^13^C.

### 2.7. Cell Culture

U87 cell line was purchased from the American Type Culture Collection (ATCC, USA) and cultured in DMEM medium supplemented with 10% (v/v) heat-inactivated FBS. Mouse splenocytes were freshly isolated from C57 mice (Central Research Laboratory, second Clinical Hospital, Changchun, China) and cultured in RPMI 1640 medium supplemented with 10% (v/v) heat-inactivated FBS and maintained at 37°C with 5% CO2 in humidified atmosphere.

### 2.8. Determination of Cell Viability

The effect of jaceosidin on cell viability was measured by MTT assay. Briefly, 5000 cells were seeded into 96 well tissue culture plates. After 24-hour incubation at 37°C, cells were treated with different concentrations of jaceosidin (30 *μ*M to 300 *μ*M) for 24 hours and 48 hours. The MTT reagent was then added to each well (500 *μ*g/mL), and the cells were further incubated for 4 h. Subsequently, 150 *μ*L DMSO was added to dissolve farmazan crystals, and absorbance was measured at 570 nm in a thermo microplate reader (Thermo Scientific, USA). Results were expressed as the percentage of MTT reduction, assuming that the absorbance of control cells was 100%.

### 2.9. Morphological Changes of U87 Cells by Light Microscopy

U87 cells were treated with jaceosidin (100 *μ*M/L) for 24 h and 48 h. Morphological changes were observed by phase contrast microscopy (Olympus 1 × 71, Japan). Freshly isolated mouse splenocytes were treated with or without jaceosidin (100 *μ*M/L) for 24 h and 48 h. After treatment, cells were stained with 0.4% trypan blue and were counted microscopically for the percentage of dead and live cells.

### 2.10. Live/Dead Cells Assay

Live and dead cells were quantified using the fluorescent probes calcein AM and PI. Calcein AM is highly lipophilic and cell membrane permeable. In viable cells, it is converted into calcein by Esterases and emits strong green fluorescence. PI, a nuclei-staining dye, cannot pass through a viable cell membrane. Cells with impaired plasma membrane integrity are stained red due to entry of PI and failure to retain calcein. Since both calcein and PI-DNA can be excited with 490 nm, simultaneous monitoring of viable and dead cells is possible with a fluorescence microscope (Olympus 1 × 71, Japan). U87 cells were incubated with or without 100 *μ*M jaceosidin for 24 h and 48 h. After incubation, floating and adherent cells were collected, washed with PBS, and incubated with PBS solution, containing 2 *μ*M calcein AM and 4 *μ*M PI in the dark for 20 min at room temperature. At the end, 100 cells were counted microscopically for the percentage of live and dead cells.

### 2.11. Flow Cytometric Analysis of Cell Death

U87 cells were treated with or without 100 *μ*M of jaceosidin, in the presence or absence of 100 *μ*M caspase 3 inhibitor, Ac-DEVD-CHO, for 24 h and 48 h. The cells were harvested, rinsed twice with PBS, and labeled with 5 uL FITC-conjugated annexin V according to the manufacturer's instructions (Beyotime, Shanghai, China). After incubation in dark for 10 min and then labeled with PI, the samples were immediately analyzed on a flow cytometer (Beckman Coulter, Epics XL).

### 2.12. Flow Cytometric Analysis of Cell Cycle

 U87 cells were treated with or without 100 *μ*M of jaceosidin for 24 h and 48 h. The cells were then washed with PBS and fixed with 70% ice-cold ethanol at 4°C for overnight. After washing twice with PBS, cells were stained with a solution containing 50 *μ*g/mL of PI and 100 *μ*g/mL RNase A for 30 min in the dark at room temperature. The stained cells were analyzed by flow cytometry (Beckman Coulter, Epics XL).

### 2.13. Determination of Reactive Oxygen Species (ROS) Generation

The changes in the intracellular reactive oxygen species generation were measured by staining the cells with 2′,7′-dichlorfluorescein-diacetate (DCFH-DA). Briefly, U87 cell were incubated in 6 well culture plates for overnight. After treating the cells with or without 100 *μ*M of jaceosidin for 24 h and 48 h, cells were further incubated with 10 *μ*mol/L DCFH-DA at 37°C for 30 min according to manufacturer's instruction (Beyotime, Shanghai, China). In the positive control group, cells labeled with DCFH-DA were treated with 1 *μ*L rose up for 30 min. Subsequently, cells were collected, rinsed, resuspended in PBS, filtered with 300 apertures and analyzed for 2′,7′-dichlorfluorescein (DCF) fluorescence by flow cytometry (FCM). Approximately, 10,000 cells were evaluated in each sample.

### 2.14. Measurement of Mitochondrial Membrane Potential Using FCM

Rhodamine 123 was used to determine the changes in mitochondrial membrane potential as described previously [10]. Briefly, U87 cells were incubated in 6 well plates for overnight. After treating the cells with or without 100 *μ*M of jaceosidin for 24 h and 48 h, cells were collected in 10 mL centrifuge tube by centrifugation at 1000 rpm for 5 min. Cells were then resuspended in 1 mL PBS solution containing 10 *μ*g Rhodamine 123 and incubated in the dark for 30 min. After incubation, cells were centrifuged at 1500 rpm for 5 min, supernatant was removed, pellet was gently rinsed with PBS once and then resuspended in 500 *μ*L PBS. After filtration (300 apertures), the suspension was analyzed by FCM.

### 2.15. Western Blot Analysis

U87 cells were treated with or without 100 *μ*M jaceosidin for indicated time period, rinsed twice with PBS, and lysed on ice with WIP cell lysis reagent (BIOSS, Beijing Biosynthesis Biotechnology Co LTD) supplemented with 1% PMSF for 30 min. The insoluble protein lysate was removed by centrifugation at 12000 rpm for 15 min at 4°C. The protein concentrations were determined using NanoDrop 1000 (Thermo Scientific, USA) spectrophotometer. 70 *μ*g of proteins were resolved on 12% SDS-PAGE and transferred to PVDF membranes. After blocking with 5% (w/v) nonfat milk and washing with Tris-buffered saline-tween solution (TBST), membranes were incubated with respective primary antibodies at 4°C for overnight and washed three times with TBST. The blots were then incubated with anti-rabbit or anti-mouse horseradish-peroxidase-conjugated secondary antibodies for 1 h. After washing with TBST three times, signals were detected using ECL plus chemiluminescence kit on X-ray film (Millipore Corporation, Billerica, USA).

### 2.16. Statistical Analysis

The results were expressed as the mean ± SEM and statistically compared with the control group or compared within the groups using one-way ANOVA followed by “Tukey's Multiple Comparison Test” and, *P* < 0.05 was considered statistically significant.

## 3. Results

**Figure 1:**
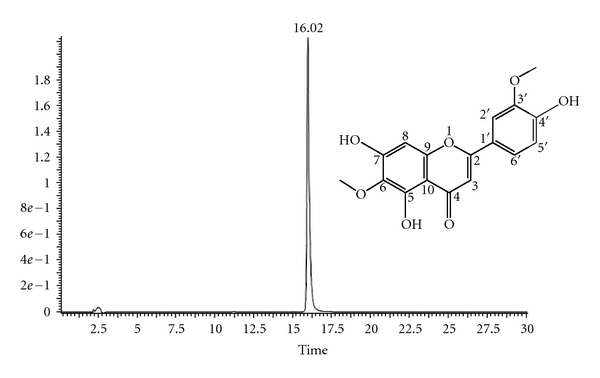
Analytical HPLC chromatogram of isolated compound (jaceosidin) and its structure. This figure shows the retain time of compound is 16.02 min and the purity is 99.5% using the HPLC method we set. Analytical reversed-phase (RP) HPLC was performed over C18 bonding chromatographic column (5 *μ*m, 2.1 × 150 mm, XTerra) and gradient elution by (A) acetonitrile: (B) 0.2% Formic acid (A: 0–25 min, 10−90%; 25–30 min, 90–100%) with flow rate 0.2 mL/min. Detection of wave length was 342 nm.

 Bioactivity-guided phytochemical study on ethanol extract of dried leaves of *Artemisia argyi* led to the isolation and purification of a flavonoid called jaceosidin. Purity of compound was assessed by analytical HPLC. Our HPLC analysis showed only one single peak at a retention time of 16.02 min (Figure 1), and purity of the peak was determined to be 99.5%. Our results intimated that jaceosidin with purity >99% could be obtained successfully by HSCCC separation under the above-optimized conditions. To the best of our knowledge, this is the first report of the isolation of jaceosidin from *Artemisia argyi* using HSCCC. The structural elucidation of HSCCC pure fraction was carried out by ESI-MS and NMR spectra as follows.

**Jaceosidin**

Yellow powder (methanol); ESI-MS m/z (%): 330 [M] +; mp: 229~230°C; molecular formula: C_17_H_14_O_7_; ^1^H NMR (500 MHz, DMSO-d6) *δ*: 3.89 (3H, s, OMe), 3.74 (3H, s, OMe), 13.07 (1H, s, 5-OH), 7.55 (2H, m, H-5′,6′), 6.93 (1H, d, *J* = 8.9 Hz, H-2′), 6.58 (1H, s, H-8), 6.87 (1H, s, H-3); ^13^C NMR (100 MHz, DMSO-d6) *δ*: 163.5 (C-2), 102.6 (C-3), 181.9 (C-4), 152.6 (C-5), 131.5 (C-6), 158.1 (C-7), 94.4 (C-8), 152.5 (C-9), 103.7 (C-10), 121.5 (C-1′), 110.2 (C-2′), 148.0 (C-3′), 150.7 (C-4′), 115.7 (C-5′), 120.4 (C-6′), 59.8 (OCH3), 55.9 (OCH3).

The MS and NMR spectral data were consistent with those reported [11]. The structure of jaceosidin is shown in Figure 1.

### 3.1. Effect of Jaceosidin on U87 Cell Proliferation

**Figure 2:**
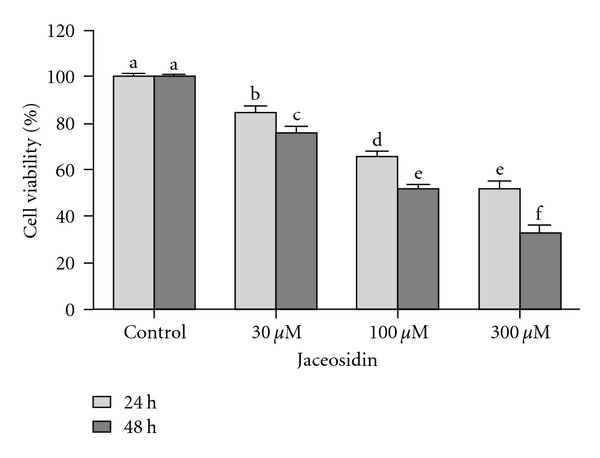
Dose- and time-dependent effect of Jaceosidin on U87 cells viability. U87 cells were treated with various doses of Jaceosidin for 24 h and 48 h. Data are expressed as Mean ± SEM of three independent experiments. Columns not sharing the same superscript letter differ significantly (*P* < 0.05).

To detect the growth inhibitory effect of jaceosidin on U87 cells, we performed MTT assay. Our results showed that jaceosidin reduced the cell viability in a time- and dose-dependent manner (Figure 2). The IC_50_ values were 300 *μ*M and 100 *μ*M after 24 h and 48 h treatment, respectively. 100 *μ*M concentration was selected for the following experiments.

### 3.2. Microscopic Study of U87 Cells

**Figure 3:**
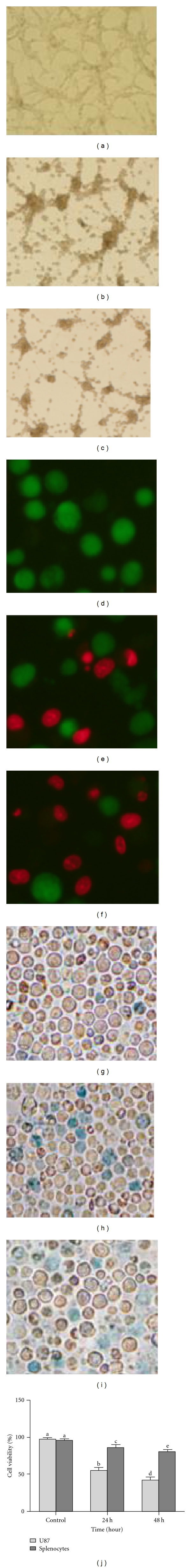
Microscopic analysis of U87 cells (a, b, c, d, e, f) and mouse splenocytes (g, h, i). (a) Untreated U87 cells; (b, c) U87 cells were treated with 100 *μ*M/L jaceosidin for 24 h and 48 h, respectively, and morphological changes were assessed by phase contrast microscopy. (d) Untreated U87 cells; (e, f) U87 cells were treated with 100 *μ*M/L jaceosidin for 24 h and 48 h, respectively. Dead cells stained red while viable cells stained green. (g) Untreated splenocytes; (h, i) splenocytes were treated with 100 *μ*M/L jaceosidin for 24 h and 48 h, respectively. Dead cells stained blue. (j) Data are expressed as Mean ± SEM of three independent experiments. Columns not sharing the same superscript letter differ significantly (*P* < 0.05).

U87 cells' morphological changes were observed with phase contrast microscopy. Cells were treated with jaceosidin at 100 *μ*M concentration for 24 h and 48 h, respectively. Treated cells displayed drastic morphological changes including a reduction in total number of cells and an increase in floating cells in culture medium in a time-dependent manner (Figures 3(b) and 3(c)).

We also examined the effect of jaceosidin on mouse splenocytes and U87 cells, using trypan blue and fluorescent probes calcein AM and PI respectively.  Figure 3 shows the staining of cells with calcein and PI. Untreated U87 cells (Figure 3(d)) took up calcein AM and deesterified and retained the green calcein dye while cells treated with jaceosidin (Figures 3(e) and 3(f)) were unable to retain the intracellular calcein or to exclude PI. Live (green) and dead cells (red) were counted microscopically. The data shows that the viability of U87 cells treated with 100 *μ*M jaceosidin for 24 h and 48 h were significantly lower (42.4% and 56.7% resp.) compared with untreated control group. However, jaceosidin exhibited less cytotoxic effect on mouse splenocytes as revealed by trypan blue dye exclusion method (Figures 3(h) and 3(i)). 

### 3.3. Jaceosidin Induces Mitotic Arrest in U87 Cells

**Figure 4:**
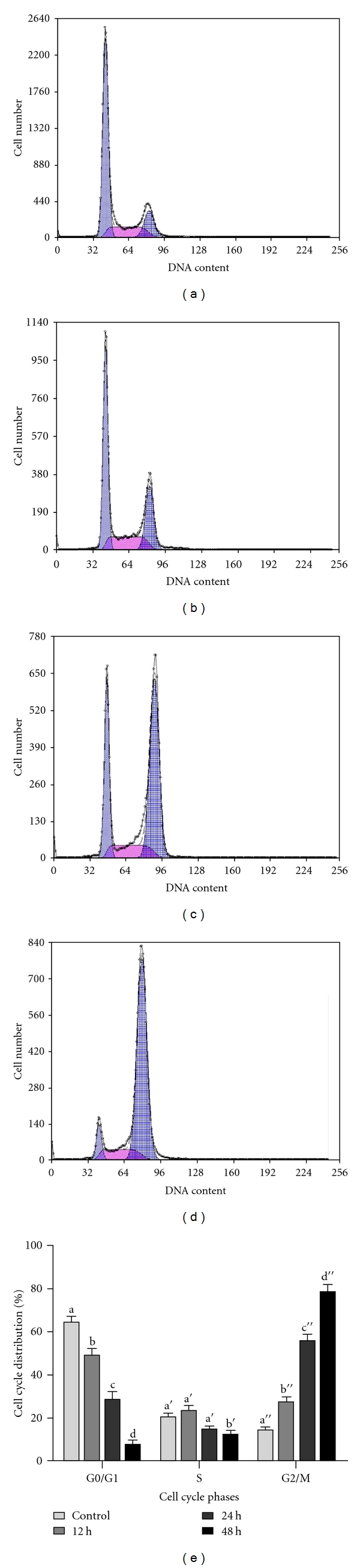
Flow cytometric analysis of the cell cycle distribution in U87 cells treated with 100 *μ*M/L jaceosidin for various times. (a) Untreated Control; (b, c, d) U87 cells were treated with 100 *μ*M/L jaceosidin for 12 h, 24 h, and 48 h, respectively. (e) Data are expressed as Mean ± SEM of three independent experiments. Columns not sharing the same superscript letter differ significantly (*P* < 0.05).

Cell cycle arrest is one of the major causes of cell growth inhibition. In order to find whether cell growth inhibition is from cell cycle arrest at a specific phase of cell cycle, cell cycle profile was analyzed using PI staining and flow cytometry analysis. Our results showed that jaceosidin arrested the cell cycle at G2/M phase in a time-dependent manner (Figure 4). Treatment with jaceosidin at 100 *μ*M showed a statistically significant increase in G2/M phase from 14.6% to 27.3%, 55.5%, and 78.6% with a concomitant decrease in G0/G1 phase from 64.3% to 49.6%, 28.4%, and 7.3% after 12 h, 24 h, and 48 h, respectively. However S phase experienced significant reduction only after 48 h treatment compared to control group.

### 3.4. Jaceosidin Induces Apoptosis in U87 Cells

**Figure 5:**
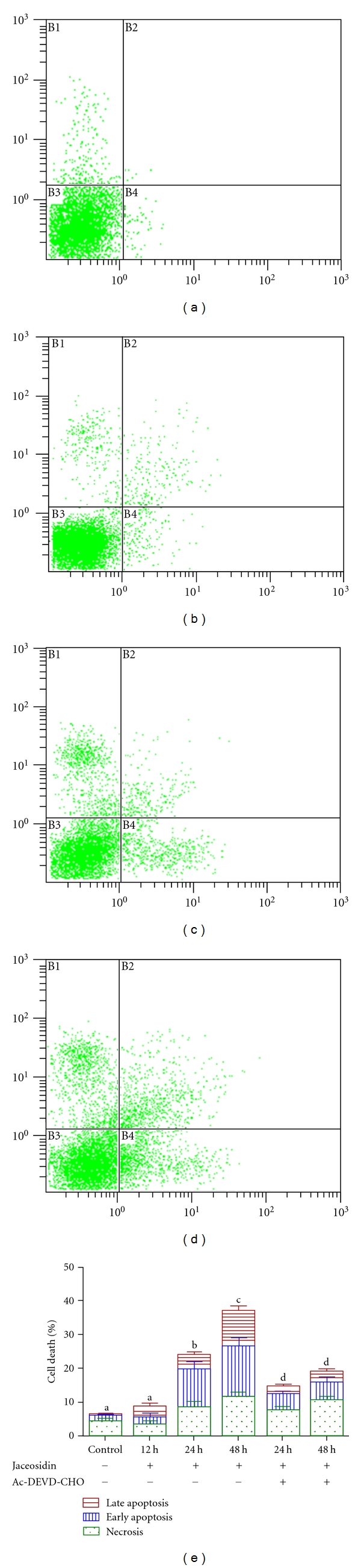
Flow cytometric analysis of apoptosis in U87 cells treated with 100 *μ*M/L Jaceosidin for various times. (a) untreated control; (b, c, d) U87 cells were treated with 100 *μ*M/L Jaceosidin for 12 h, 24 h, and 48 h, respectively. (e) Cells were treated with or without 100 *μ*M/L Jaceosidin in the presence or absence of 100 *μ*M Ac-DEVD-CHO for various times. Data are expressed as Mean ± SEM. of three independent experiments. Columns not sharing the same superscript letter differ significantly (*P* < 0.05).

To further investigate jaceosidin-induced inhibitory effect, U87 cells were treated with jaceosidin as described in Materials and Methods section and the percentages of cells undergoing apoptosis/necrosis were determined by flow cytometric analysis after staining with annexin V-FITC and PI. Our results showed that jaceosidin caused a time-dependent apoptosis. A significant increase was observed in both early and late apoptosis in 48 h treatment group whereas 24 h treatment group showed significant increase only in early apoptosis as compared with control group (Figure 5). However, no significant apoptosis has been observed in 12 h treatment group.

Furthermore, there was significant decrease in both early and late apoptosis, when the cells were treated with 100 *μ*M Ac-DEVD-CHO, a caspase 3 inhibitor (Figure 5(e)). The data suggested that jaceosidin induces caspase-3-dependent apoptosis in U87 cells.

### 3.5. Jaceosidin Induces ROS Production in U87 Cells

**Figure 6:**
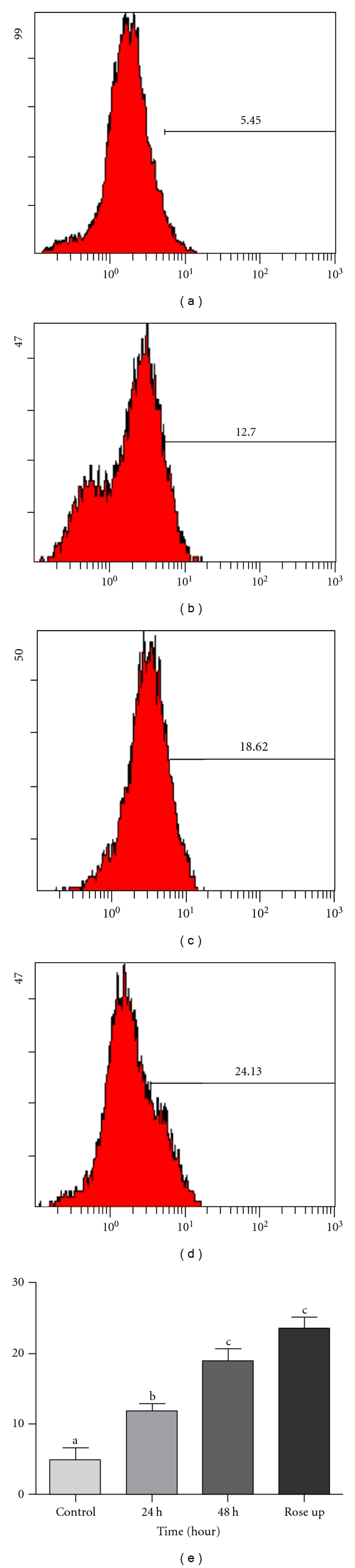
The level of ROS in U87 cells treated with 100 *μ*M/L jaceosidin for various times. (a) Untreated control; (b, c) cells were treated with 100 *μ*M/L jaceosidin for, 24 h and 48 h, respectively; (d) Rose up; (e) Data are expressed as Mean ± SEM of three independent experiments. Columns not sharing the same superscript letter differ significantly (*P* < 0.05).

Flavonoids are strong antioxidant agents and increase intracellular ROS production. So, we measured the level of ROS in treated and untreated groups by staining cells with DCFH-DA, using FCM. Our results showed that jaceosidin increased the level of ROS from 4.91% to 11.9% and 19.1% after 24 h and 48 h treatment, respectively. The level of ROS in positive control group (Rose up) was 23.5% (Figure 6).

### 3.6. Effect of Jaceosidin on Mitochondrial Membrane Potential

**Figure 7:**
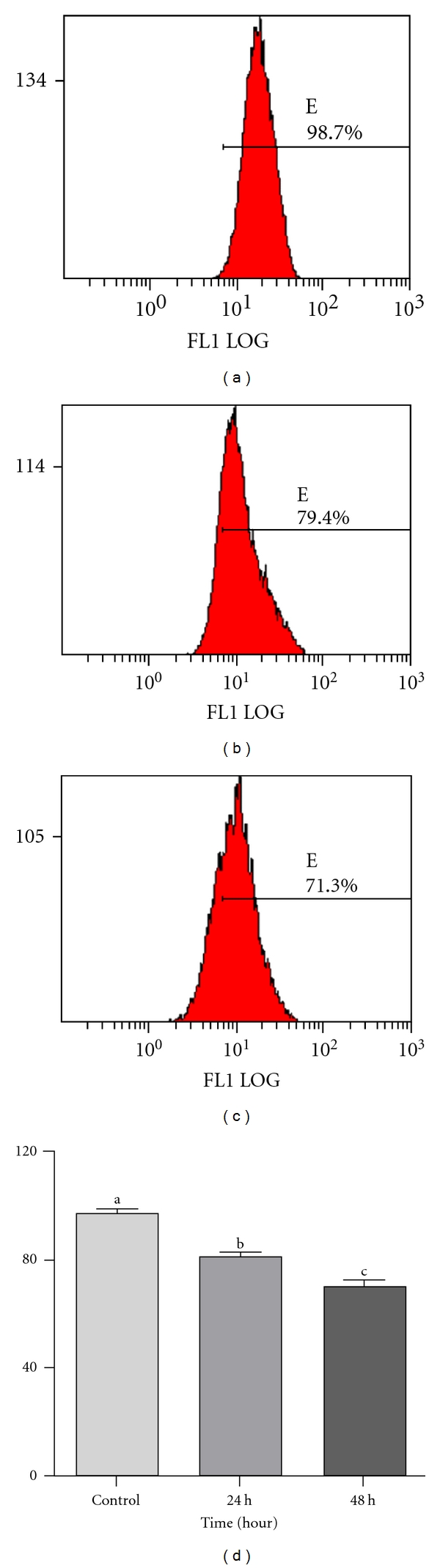
The mitochondrial membrane potential in U87 cells treated with 100 *μ*M/L Jaceosidin for various times. (a) Untreated control; (b, c) Cells treated with 100 *μ*M/L Jaceosidin for 24 h and 48 h, respectively; (d) Data are expressed as Mean ± SEM. of three independent experiments. Columns not sharing the same superscript letter differ significantly (*P* < 0.05).

Depolarization in mitochondrial membrane potential is a characteristic feature of apoptosis. We determined mitochondrial membrane potential in treated and control groups using FCM. Our results showed that mitochondrial membrane potential in U87 cells treated with 100 *μ*M jaceosidin was 80.3% and 69.6% after 24 h and 48 h, respectively. These values were significantly lower than those of control group (97%) as shown in Figure 7.

### 3.7. Effect of Jaceosidin on the Expression of Apoptosis Regulators

**Figure 8:**
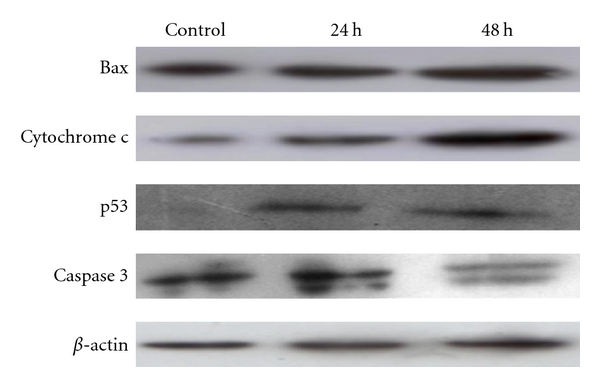
Effect of jaceosidin on the expression of the apoptosis regulators. U87 cells were treated with 100 *μ*M/L jaceosidin for 24 h and 48 h, respectively. The levels of Bax, Cytochrome c, p53, and caspase 3 were determined by Western blot analysis.

To investigate the mechanism of apoptosis in U87 glioma cells mediated by jaceosidin, expressions of some major apoptosis regulatory proteins (p53, Bax, cytochrome c, and caspase 3) were detected by Western blot. As shown in Figure 8, the expression level of p53 and Bax was markedly increased by jaceosidin accompanied with the release of cytochrome c from mitochondria to cytosol and cleavage of caspase 3 in a time-dependent manner. These results together revealed that jaceosidin induces apoptosis in U87 glioma cells through intrinsic pathway.

### 3.8. Jaceosidin Induces Apoptosis-Independent Cell Cycle Arrest in U87 Cells

To characterize in detail the relationship between cell cycle arrest and apoptosis mediated by jaceosidin in U87 cells, we performed flow cytometric analysis of apoptosis and cell cycle arrest after 12 h treatment with jaceosidin. As shown in Figure 4(b), there was a statistically significant increase in G2/M phase with a concomitant decrease in G0/G1 phase (*P* < 0.05) of cell cycle after 12 h treatment, whereas no increase was observed in apoptosis rate after 12 h treatment (Figure 5(b)) which showed that cell cycle arrest is an early event in U87 cell growth inhibition induced by jaceosidin. These results were further confirmed by using a specific caspase 3 inhibitor, Ac-DEVD-CHO. Flow cytometric analysis of U87 cells treated with 100 *μ*M jaceosidin in the presence and absence of (100 *μ*M) caspase 3 inhibitor, Ac-DEVD-CHO was performed. Ac-DEVD-CHO significantly (*P* < 0.05) inhibited apoptosis but did not prevent the mitotic cell cycle arrest, indicating that cell cycle arrest occurs independently of apoptosis and is an early event in cell death mediated by jaceosidin (data not shown). These results together insinuated that jaceosidin detained the cell cycle at G2/M phase and thereby induces apoptosis.

## 4. Discussion

Cell cycle control is one of the major regulatory mechanisms of cell growth [12–15]. Many anticancer agents have been reported to arrest the cell cycle at a specific checkpoint and thereby induce apoptotic cell death [12, 14, 16, 17]. One of the checkpoints, the G2/M checkpoint arrests the cell cycle at G2 phase when DNA is damaged [18, 19]. P53 tumor suppressor protein plays a key role in the regulation of cell cycle and cell death. Activation of p53 may lead to growth arrest at G1 or G2 phase of the cell cycle and apoptotic cell death by multiple pathways [20–23]. In the present study, we showed that jaceosidin is able to inhibit growth of U87 glioblastoma cells. However, this growth inhibition might be resulted from apoptosis, necrosis, or cyclic block [24]. To further investigate whether this effect was from apoptotic induction or cell cycle arrest, flow cytometric analysis of cell cycle and apoptosis was conducted. The flow cytometric analysis of cell cycle showed that jaceosidin induced cell cycle arrest at G2/M phase accompanied by a reduction in G0/G1 phase in a time-dependent manner. The tumor suppressor protein p53 has been reported previously to play a critical role in G2/M arrest and G0/G1 arrest [25]. Because U87 cells express wild type p53, we examined possible changes in the protein expression induced by jaceosidin treatment. We found that p53 expression increased dramatically in the cells treated with jaceosidin in a time-dependent manner.

Many cytotoxic agents arrest the cell cycle at G0/G1, S, and G2/M phase and then induce apoptosis [11, 13, 15, 16]. So next, we performed flow cytometric analysis of apoptosis by FITC-labeled annexin-V and PI staining to check whether jaceosidin could induce apoptosis in U87 cells. The results demonstrated that jaceosidin induced significant apoptosis in U87 cells after 24 h and 48 h treatment. Previous studies have shown that jaceosidin induced apoptosis in *ras*-transformed human breast epithelial cells through generation of reactive oxygen species [8]. We here examined whether jaceosidin increases the intracellular level of ROS in U87 cells. The data showed that jaceosidin increased the level of ROS in U87 cells in a time-dependent manner. However, pretreatment with NAC did not prevent jaceosidin induced apoptosis and cell cycle arrest at G2/M phase, indicating that ROS generation is not involved in jaceosidin-induced apoptosis in U87 cells.

To check whether apoptosis is dependent on cell cycle arrest, we performed flow cytometric analysis of cell cycle and apoptosis after 12 h treatment with jaceosidin. The data showed that 12 h jaceosidin treatment significantly arrested the cell cycle at G2/M phase, however no significant increase in apoptosis rate was observed. Furthermore, inhibition of caspase 3 activation with Ac-DEVD-CHO attenuated apoptosis but did not prevent mitotic arrest indicating that apoptosis occurs downstream of the cell cycle arrest. To the best of our knowledge, this is the first report presenting that jaceosidin induced apoptosis through cyclic block.

It has been reported that p53, a tumor suppressor protein, triggers apoptosis by inducing mitochondrial outer membrane permeabilization through Bax upregulation and Bcl-2 downregulation [20, 26, 27]. To gain further insights into the molecular mechanism underlying jaceosidin-induced apoptosis, we examined expression of Bax protein in cells of each group. The result indicated that expression of Bax gradually increased in treatment groups in a time-dependent manner. Involvement of mitochondrial pathway in Bax-mediated apoptosis was further confirmed by observing changes in mitochondrial transmembrane potential (MTP) using flow cytometry. A significant reduction in mitochondrial membrane potential has been observed in the cells of treatment groups, suggesting the opening of mitochondrial permeability transition pores (PTP). Therefore, we concluded that jaceosidin can promote the opening of mitochondrial PTP by increasing the Bax/Bcl-2 ratio. 

The opening of mitochondrial PTP can lead to the release of cytochrome c and other proapoptotic molecules from intermembrane space to cytosol where cytochrome c binds and activates caspase 9 which then leads to the activation of other downstream caspases and ultimately caspase 3. Caspase 3 has been identified as a key mediator of apoptosis of mammalian cells [10, 28].

We next investigated the effect of jaceosidin on the release of cytochrome c and activation of caspase 3 in U87 cells. As shown in Figure 8, jaceosidin promoted the release of cytochrome c and cleaved caspase 3 in a time-dependent manner. These results are in line with those reported previously [8, 9]. Furthermore, treatment of U87 cells with caspase 3 inhibitor, Ac-DEVD-CHO, resulted in a significant decrease in apoptosis indicating caspase 3 is involved in apoptosis induced by jaceosidin. These results further confirmed that jaceosidin induced apoptosis in U87 cells through mitochondrial-caspase-3-dependent pathway. However, in the present study, the mechanism of p53 activation by jaceosidin remains unclear. We are continuing our further studies to investigate the mechanism of p53 activation by jaceosidin.

 In conclusion, our data demonstrated that jaceosidin inhibited the growth of U87 glioblastoma cells and induced apoptosis through cell cycle arrest, upregulation of p53 and Bax, lowering MTP, release of cytochrome c, and cleavage of caspase 3. Thus, jaceosidin could be developed into a novel chemotherapeutic or chemopreventive agent against glioblastoma.

##  Conflict of Interests

The authors have declared that no conflict of interests exists.
